# Supplementary material for: Are carbon emissions trading and green financial instruments synergistic? -Comprehensive quantitative research based on content analysis
Source: PLoS One. 2024 Mar 7;19(3):e0298601. doi: 10.1371/journal.pone.0298601 (PMC10919601; doi:10.1371/journal.pone.0298601)
Supplement: S1 Data — (ZIP) [file pone.0298601.s001.zip › Data/地级市Z_F工作报告.zip!/╔╜╬≈/┴┘╖┌╩╨/└┤╫╘╣1⁄2╓┌║┼í╛┬φ┐╦╩2╛▌═°í┐_2016.docx]

各位代表：

　　现在，我代表市人民政府向大会报告工作，请予审议，并请市政协委员和其他列席人员提出意见。

　　一、十二五”时期工作回顾

　　“十二五”时期是我市发展历程中极不平凡的五年。五年来，在市委的坚强领导下，在市人大、市政协的监督支持下，市政府紧紧依靠全市人民，认真贯彻落实党的十八大、十八届三中、四中、五中全会精神和习近平总书记系列重要讲话精神，按照“四个全面”战略布局和省委“五句话”要求，全面从严治党，深入推进党风廉政建设和反腐败斗争，加快实施“六权治本”，着力推动“六大发展”，扎实推进“稳增长、调结构、惠民生、防风险”各项工作，积极应对挑战，奋力攻坚克难，不断开创弊革风清、富民强市新局面，全市经济社会发展取得了新的成就。

　　经济发展稳中有进。2015年，全市地区生产总值完成1161.1亿元，“十二五”期间年均增长7.6%;规模以上工业增加值完成334.4亿元，年均增长8.6%;固定资产投资完成1401.2亿元，年均增长24%;一般公共预算收入完成88.2亿元，年均增长3.%;社会消费品零售总额完成572亿元，年均增长12.6%;城镇居民人均可支配收入25498元，年均增长11%;农村居民人均可支配收入9376元，年均增长11.9%。我市综合经济实力迈上了新台阶。

　　产业结构更趋合理。现代农业稳步推进。粮食总产达到23.亿公斤，比“十一五”末增长22.%。“四个百万亩”基地建设成效显著，新增设施蔬菜12万亩、水果42万亩、干果35万亩、中药材90万亩。2015年，全市农民合作社达到10059家，农业产业化龙头企业发展到339家，农产品年销售收入达到73亿元，是“十一五”末的1.4倍。传统产业实现优化升级。煤、焦、冶、电等传统产业整体素质进一步提升，装备制造、铸造、现代煤化工、新能源、新型材料、电子信息等战略性新兴产业加快发展，共实施新兴产业项目330个，完成投资814.3亿元，占全市工业领域总投资的67%。现代服务业蓬勃发展。商贸物流、电子商务、文化旅游等现代服务业不断发展壮大。建成运营居然之家、生龙国际、嗨都国际广场等一批商贸转型项目。我市被确定为全国区域级流通节点城市。全市电商企业发展到400余家，交易额突破100亿元，侯马开发区被确定为“国家电子商务示范基地”。大力推进旅游景区建设，累计完成投资52亿元，全市新增5个国家4A级旅游景区，总数达11个，位居全省第一。2015年，全市旅游总收入达到295亿元，比“十一五”末增长152.9%。全市三次产业结构由7.∶58.3∶34.2优化为7.8∶48.5∶43.7，现代产业体系正在加快形成。

　　改革开放不断深化。行政审批制度改革迈出重大 步伐，累计承接、取消、下放、调整行政审批项目290项，行政审批时限压缩了三分之二，市县两级权责清单全部公布。建成市政务服务中心，实行“一委一办三中心”的管理体制。完成了煤炭焦炭公路销售体制改革，所有煤焦站点和涉煤票据全部清理取消。商事登记制度、医疗卫生体制、国有林场、财税体制、投融资体制、国有企业、集体林权配套制度、开发区扩区等各项改革都取得了积极成效。对外开放水平进一步提升，实际利用外资由7757万美元增长到15660万美元，年均增长15.1%。在全省率先启动招商引资2.0模式，“十二五”期间累计签约项目899个，签约资金14319亿元，是“十一五”的4.6倍。

　　项目建设力度空前。深入开展了“项目落地年”“项目推进年”“项目见效年”和“项目提质增效年”等活动，坚持统筹推进重点项目建设，“六位一体”各项指标连年位居全省前列。五年累计实施省市县重点项目4116项，完成投资6053亿元，超额完成“五年五千亿”的目标任务，投资额是“十一五”时期的3.6倍，为我市经济社会发展奠定了坚实的基础。全市公路通车里程达到18245公里，位居全省第一。新改建干线公路797公里、农村公路3135公里。新增高速公路通车里程267公里，临汾北环、临吉高速、霍永高速等一批高速公路建成通车。大西高铁、中南铁路开通运营，临汾民航机场正式通航。城镇化建设步伐加快，“一城三区”、侯马曲沃同城化、12个大县城和60个重点镇建设扎实推进。全市启动实施城中村改造工程47个，新建城市公厕318座。市区先后实施了22条城市主干路和古城公园二期、市规划展览馆、大西客运站站前广场等市政工程;全面铺开了总投资159.9亿元的市区17项重点城建工程，城市管理全面加强，城市功能进一步完善。引沁入汾和川取水输水工程、五马水库、清峪水库等项目顺利完工，14条中小河流综合治理和45座病险水库除险加固工程全面完成，水利建设总投资达到101亿元，是“十一五”的3.9倍。

　　“百里汾河生态经济带”建设取得突破。“经济带”总体规划纳入《晋陕豫黄河金三角区域合作发展规划》，获国务院批复，6个专项规划通过国家级专家评审。设立了百里汾河生态文明保护区，加强了汾河流域生态保护。投资42亿元的滨河东路贯通工程通车运行，投资43亿元的汾河河道生态治理工程全面竣工，投资58亿元的涝洰河生态建设工程初见成效。打造了“百里汾河文化长廊”，规划建设了汾河非遗生态保护区。“经济带”内18个工业园区的销售收入占到全市规模以上企业销售收入的63%，8个现代农业园区初具规模，5个物流园区开工建设，17个文化旅游景区全部实施开发，11个“两区同建”工程进展顺利。“百里汾河生态经济带”已成为我市加快发展的新引擎，展现出良好的发展活力和前景。

　　“三个突破”深入推进。金融振兴方面，积极引进培育金融机构，全市银行业金融法人机构达到23家、保险经营主体33家、证券分支机构5家、各类交易市场4家，初步形成了银行、证券、保险、产权交易等各业并举、功能基本完备的金融体系。积极培育企业上市，在“新三板”上市企业达到5家，总量居全省第二;在山西股权交易中心挂牌企业达到143家，占到全省总量的11%。积极破解企业融资难题，设立了5000万元的“企业应急周转保障资金”，引导筹集1.56亿元的“企业应急周转互助资金”，累计投放金额8.7亿元。市投资集团公司累计融资244.8亿元。科技创新方面，共实施国家、省、市科技计划项目418项，累计投入科技研发经费1.4亿元。培育省级科技示范园区2个，市级科技示范基地34个;建成院士工作站2个，博士后流动工作站1个。全市高新技术企业达到33家，其中国家级企业技术中心1家，省级企业技术中心20家，省级行业技术中心1家。全市专利拥有量达到329件。民营经济发展方面，出台了《进一步支持民营经济健康发展的措施》，共创办小微企业9000家，培育“小巨人”企业42家、“小升规”企业69家，建成5个省级中小企业创业基地。2015年，全市中小企业工业产品销售产值达到682亿元，占到全市工业产品销售产值的54.%，民营经济成为拉动我市经济增长的主力军。

　　生态环境持续改善。环保工作再上新台阶，持续开展环境建设年、环境提升年等活动，累计投资15.5亿元，实施了169个环保项目。完成5家电力企业脱硫脱硝治理、16家焦化企业焦炉烟气治理，淘汰黄标车及老旧车5.12万辆。成功创建省级环保模范城市。2015年，市区空气质量二级以上天数达到266天，按新标准比2013年增加99天，PM2.浓度比2013年下降21.3%。全市新建垃圾处理场14座，实现生活垃圾处理场县级全覆盖。汾河出境断面主要污染物化学需氧量和氨氮浓度低于入境断面。全市饮用水水源地水质达标率保持在100%。节能减排呈现新亮点，五年累计实施节能改造项目288个，累计淘汰落后产能1675.2万吨。全市新增和更新新型环保公交车580辆，其中2015年市区新购纯电动车223辆。我市入选“国家节能减排财政政策综合示范城市”，尧都区城东老工业区搬迁改造纳入国家试点，浮山县入选国家资源综合利用“双百工程”示范基地，古县入选首批省级低碳县试点。生态建设取得新进展，五年营造林246万亩，森林覆盖率由27.47%增长到31.91%。扎实推进水土保持重点工程，累计完成治理面积214万亩。持续推进采煤沉陷区、采空区、水土流失区、煤矸石山等生态环境治理修复工程，西山7县成为国家主体功能区建设试点。

　　安全生产全面加强。全面推进安全生产责任体系“五个全覆盖”，组织开展了“安全生产稳定年”“安全生产强基年”和“知责、履责”“打非治违”等活动，持续开展安全生产大检查，切实加强重点行业和领域安全监管，强化安全检查全留痕、企业ABCD分级分类监管和企业安全生产标准化建设等安全管理措施。2015年，全市安全生产事故总量和死亡人数比“十一五”末分别下降14.5%、25.8%，工矿企业连续7年未发生重特大事故，安全生产形势实现持续稳定好转。全面加强信访、综治、应急、舆情监控等工作，建立了市级领导包联信访事项制度，开通了“市长热线”“市长信箱”，完成了“六五”普法任务，全面推广安泽“311”基层服务管理、乡宁“三化一卡”、侯马“345”流动人口管理、铁路安全“线格化”管理等社会管理先进经验，社会保持和谐稳定。

　　民生质量显著提高。文化方面，完成17个县(市、区)的文化馆、图书馆标准化建设，新建临汾市博物馆、晋国博物馆、丁村民俗博物馆等16个博物馆。全市有300多件文化作品获“山西广播电视奖”和“全省优秀广播电视栏目奖”。教育方面，投资4.亿元的临汾新 高中建成投用，市第三中学、市第一小学等市直学校改造和17个县(市、区)义务教育薄弱学校改造工程全面完成。新改扩建公办标准化幼儿园144所、农村幼儿园134所。2015年高考达二本线16189人，比2010年增加7112人，达线率上升19.1个百分点。职业教育、成人教育和民办教育全面发展，教育教学质量稳步提升。卫生方面，临汾新医院和10所县级医院建成运营，全市医院总数达到116家，医疗卫生机构床位数总量达到13805张，分别比“十一五”末增长20.%和22.3%。新创建“三甲”医院3家，创建省级以上卫生县城15个。新农合参合率达到98.7%，人均筹资水平提高到470元。县级公立医院改革实现全覆盖。社保方面，五大社会保险参保人数达477万人次，是“十一五”时期的2.5倍。城乡低保实现应保尽保。开工建设各类保障性住房78725套，完成农村危房改造48232套。城镇登记失业率稳定控制在3%以内。我市被确定为“国家信息惠民试点城市”。扶贫方面，大力推进扶贫开发，实现脱贫25万人。重点推进光伏扶贫，实施了127个村级光伏电站和9个10MW以上地面小型电站工程，建设总规模达到134.5MW，走在了全省、全国前列。圆满完成新一轮农村“五个全覆盖”和农村“五件实事”。大力推进城市和农村人居环境改善工程。全市新增供水管网280公里，城镇供水普及率达到99%;新增集中供热面积1539万平方米，城镇供热普及率达到78.5%;新建燃气管网1012公里，城镇燃气普及率达到85%;完成了市区10万户天然气置换。连续两年实施“六大惠民工程”，群众得到了更多的实惠。

　　积极推进军民融合发展，国防动员和国防后备力量建设得到加强。民族宗教、外事侨务、老龄、残疾人、红十字会、妇女儿童等事业都有了新进步，人防、统计、工商、质监、地方志、气象、档案、地震、住房公积金等工作均取得新进展。

　　在推进经济社会发展的同时，我们始终把建设人民满意政府作为努力方向，不断加强法治政府、廉洁政府和服务型政府建设。坚持向人大及其常委会报告工作，向人民政协通报情况，主动自觉接受监督。五年共办理市人大代表建议717件、市政协委员提案1326件。坚持依法行政，完善政府决策规则和程序，深入推进政务公开。认真落实中央“八项规定”，扎实开展党的群众路线教育实践活动、学习讨论落实活动和“三严三实”专题教育，“文风会风”持续改善，“三公”经费连年下降，公务用车制度改革顺利推进。全面落实政府系统党风廉政建设责任制，切实加强“一岗双责”，深入推进“六权治本”，不断加大监察和审计工作力度，从严从实整治“四风”和不作为、懒作为、乱作为等突出问题，政府廉政建设得到进一步加强。

　　刚刚过去的2015年，是改革发展稳定任务非常繁重的一年。一年来，我们认真落实中央、省一系列政策措施，坚持抓减负保增长、抓生产保产值、抓资金保运行、抓销售保市场、抓合作促共赢，实行周报告、月调度、合署办公、督查约谈，狠抓项目建设，全力以赴保增长，实现了经济持续健康发展和社会和谐稳定，开创了弊革风清、富民强市的新局面。2015年，我市5大类52项年度目标考核任务，除地区生产总值、一般公共预算收入、规模以上工业增加值三项预期性指标因市场变化没有完成外，其余指标全面完成。

　　回顾这五年，我们深切体会到，推动临汾经济社会持续平稳发展，实现富民强市，必须坚持全面从严治党，加强党风廉政建设和反腐败斗争，坚决落实“两个责任”，把纪律和规矩挺在前面;必须坚持发展第一要务，主动适应经济新常态，转变发展方式，优化经济结构，注重发展的质量和效益;必须坚持改革创新，解放思想、求新求变，在抢抓机遇中走在前列，形成引领发展、率先发展的强大势能;必须坚持底线思维，牢牢抓住安全生产、环境保护等关乎全局的重点工作，为经济社会发展营造良好的环境;必须坚持以人为本，切实保障和改善民生，让人民群众得到更多的实惠，增强群众的获得感，不断开创经济社会发展新局面。

　　各位代表，我市“十二五”时期改革发展成就来之不易，这是党中央、国务院、省委、省政府和市委正确领导的结果，是市人大、市政协大力支持、有效监督的结果，是全市广大干部群众攻坚克难、开拓奋进的结果。在此，我代表市政府，向全市人民，向人大代表、政协委员，向驻临部队、武警官兵和所有关心支持临汾改革发展的各界朋友，表示崇高的敬意和衷心的感谢!

　　在肯定成绩的同时，也应该清醒地认识到，我市经济社会发展中仍然存在不少困难和问题，主要是：经济下行压力依然较大，企业仍然面临诸多困难;传统产业升级改造步伐不快，产能过剩问题亟待化解;经济发展的质量和效益不高，转型发展任务艰巨;自主创新能力不强，发展后劲不足;区域发展不平衡问题仍然突出，民生社会事业存在薄弱环节，脱贫攻坚任务艰巨;城市建设相对滞后，基础设施建设亟待加强;安全生产基础仍不牢固，生态环境压力较大;发展环境需要优化，作风建设任重道远，等等。对此，市政府将本着对人民高度负责的精神，以“三严三实”的作风，采取积极有效的措施，加快解决这些问题。

　　二、十三五”时期经济社会发展主要目标和任务

　　根据市委三届七次全会精神及“十三五”规划《建议》，市政府编制了“十三五”规划《纲要(草案)》，提交大会审查。

　　《纲要(草案)》按照全面建成小康社会的目标要求，强调要牢固树立和贯彻落实创新、协调、绿色、开放、共享的发展理念，全面推进创新发展、协调发展、绿色发展、开放发展、共享发展、廉洁和安全发展，明确了今后五年经济社会发展的总体目标和主要任务，提出要把临汾建设成为中西部经济强市、旅游强市、生态强市。

　　经济强市就是要推动我市经济持续平稳较快发展，新旧发展动能接续转换，创新能力显著增强，产业结构优化升级，投资效率和企业效益明显提升。重点实施投资112亿元的沃特玛新能源汽车核心技术产业园、投资145.5亿元的汾西其亚年产240万吨氢氧化铝及60万吨高精铝板带箔、投资30.2亿元的临汾空港仓储物流园等一批带动能力强的重大产业项目。全市战略性新兴产业占比达到10%以上，全员劳动生产率年均增长7%左右，主要经济指标人均水平进入中西部地级市前列。

　　旅游强市就是要加快旅游景区景点建设，形成“核心突出、布局合理、业态多元、协调发展”的旅游产业体系，基本建成全国著名旅游目的地，成功申报国家级历史文化名城。建设投资26亿元的陶寺遗址保护利用项目。实施“旅游畅通”工程，规划建设投资65亿元的霍州—— —侯马城际轻轨、投资33亿元的环城高架等一批重大基础设施项目，投资39亿元，新建改建旅游专线公路655公里。旅游总收入年均增长20%以上，旅游业增加值占地区生产总值的比重达到15%，旅游业成为全市综合性战略支柱产业。

　　生态强市就是要高标准完成国家节能减排财政政策综合示范、资源节约和循环利用、低碳经济发展等国家和省试点任务，打造国家级光伏发电示范基地和全国电动公交绿色出行示范城市。大气、水、土壤污染治理取得新成效，生产方式和生活方式绿色低碳化水平显著提高。加强沿黄地区水土保持和生态修复，加快推进投资452亿元的黄河古贤水库、投资260亿元的西山七县主体功能区建设等工程，构筑吕梁山生态脆弱区、太行山水源涵养、百里汾河经济带湿地植被恢复三大生态屏障。全市生态环境持续改善，森林覆盖率、城市建成区绿化率明显提高。创建国家环保模范城市、国家级园林城市。

　　围绕这一总体目标，今后五年，全市经济社会发展的主要任务是：

　　--经济综合实力实现新提升。到2020年，地区生产总值和城乡居民人均收入比2010年翻一番。地区生产总值达到1630亿元，年均增长7%，城乡居民收入分别达到35000元、13150元，年均增长6.5%和7%;每年减少贫困人口6万人以上，到2020年末，全市现有26.5万贫困人口全部实现脱贫;一般公共预算收入年均增长6.5%;规模以上工业增加值年均增长8%;社会消费品零售总额年均增长10%。

　　——转变发展方式取得新突破。实施创新驱动发展战略，加快新旧发展动能接续转换，着力推进供给侧结构性改革，提高供给体系质量和效率，提高全要素生产率，努力实现更高质量、更有效率、更加公平、更可持续的发展。

　　——推进改革开放形成新格局。资源型经济转型综合配套改革取得重大进展，重点领域和关键环节改革取得成效，支撑资源型经济转型的政策体系和体制机制基本建立，技术创新体系、金融支持体系、平台带动体系、政策服务体系全面完善。开放型经济和对外合作体制基本形成，对外开放的广度和深度不断拓展。

　　——文化强市建设得到新发展。文化强市建设步伐进一步加快，文化发展主要指标、文化事业整体水平、文化产业综合实力明显提升。实施精品培育战略，搭建文艺作品创作和展示平台，创作生产更好更多的优秀文艺作品，促进全市文化大繁荣。优化文化产业布局，建设文化产业基地、园区和特色产业群，推动文化产业融合性发展，使文化产业成为国民经济支柱性产业。创新公共文化服务，丰富群众文体活动，到“十三五”末基本建成上下联动、覆盖城乡的公共文化服务体系。

　　——保障改善民生达到新水平。就业持续增加，教育现代化水平显著提高，劳动年龄人口受教育年限稳步提升。社保、医疗、住房等公共服务体系更加健全，基本公共服务均等化程度显著提高。

　　——民主法治建设再上新台阶。人民民主不断扩大，法治政府基本建成。服务型政府建设成效显著，政府公信力和行政效率进一步提高。民主法制更加健全，社会治理能力和水平不断提高，社会更加和谐稳定。

　　各位代表，《纲要(草案)》经本次会议审议通过后，我们将全力抓好落实。只要全市上下同心协力，真抓实干，开拓创新，攻坚克难，经过五年的努力，一个经济更加繁荣、社会更加和谐、生态更加优美、人民更加幸福的临汾必将呈现在平阳大地!

　　三、2016年工作安排

　　今年是全面建成小康社会决胜阶段的开局之年，也是推进结构性改革的攻坚之年，做好今年的政府工作十分重要。

　　今年政府工作的指导思想是：高举中国特色社会主义伟大旗帜，全面贯彻党的十八大、十八届三中、四中、五中全会精神，以邓小平理论、三个代表”重要思想和科学发展观为指导，深入贯彻习近平总书记系列重要讲话精神，按照“五位一体”总体布局和“四个全面”战略布局，认真落实省委、省政府和市委的决策部署，坚持改革开放，坚持以新发展理念引领发展，坚持稳中求进工作总基调，适应经济发展新常态，着力加强供给侧结构性改革，扎实做好“三去一降一补”工作，努力实现“十三五”时期经济社会发展良好开局。

　　今年经济社会发展的主要预期指标是：地区生产总值增长6.5%左右，规模以上工业增加值增长3%，全社会固定资产投资增长12%，社会消费品零售总额增长6%，城镇居民人均可支配收入增长6.5%左右，农村居民人均可支配收入增长7%以上，一般公共预算收入下降7%，居民消费价格涨幅控制在3%左右。约束性指标：万元地区生产总值能耗下降4.22%，其他约束性指标按照国家节能减排财政政策示范城市的要求及省下达任务全面完成。

　　确定地区生产总值增长6.5%左右，主要是考虑与全面建成小康社会目标相衔接，考虑经济下行压力和推进结构性改革。全社会固定资产投资增长12%，主要是考虑发挥投资的关键作用，促进投资合理增长和结构优化。城镇居民人均可支配收入增长6.5%左右，农村居民人均可支配收入增长7%以上，体现了居民收入增长与经济发展同步的要求、缩小城乡收入差距的要求。一般公共预算收入下降7%，充分考虑了结构性减税、企业减负、非税收入减少等因素。综合分析各方面情况，今年我市发展面临的困难更多、挑战更为严峻，我们要做好打硬仗的充分准备，要抢抓机遇，主动作为，积极进取，狠抓落实，“跳起来摘桃子”，圆满完成今年的各项目标任务。重点要抓好以下工作： (一)积极主动作为，推动经济持续平稳发展

　　精准帮扶实体企业。进一步强化“五抓五保”措施，加大市县领导包联企业力度。认真落实国家、省里和我市的一系列惠企政策，建立完善涉企收费清单制度，切实减轻企业负担。引导企业加强内部管理，削减非生产性开支，降低人工、物流、财务等综合成本。推动煤电联营、煤化联营、煤焦钢联营，支持企业“抱团取暖”，增强抵御市场风险的能力。积极开展政银企对接，用好用活“过桥资金”，政府采购优先使用本地产品，帮助企业融通资金、开拓市场、稳定生产。全市原煤产量达到7000万吨、焦炭达到1800万吨、生铁达到1100万吨、钢材达到1200万吨、发电量达到210亿度。

　　切实抓好项目建设。紧紧围绕省“四个方面、十大领域”投资方向，完善双包双联、用地专供、联动建设、每月专题调度等项目建设机制。全年项目储备完成20000亿元，签约2000亿元，落地1510亿元，开工1520亿元，建设1300亿元，投产1320亿元，全社会固定资产投资完成1570亿元。创新投融资机制，完善政府和社会资本合作模式，鼓励社会资本参与基础设施、公共服务设施等建设。加大招商引资力度，创新招商方式，继续推进招商引资2.0模式，大力开展精准招商，积极组织自主招商活动，努力引进更多的大项目、好项目。

　　打造经济增长新引擎。持续推进“百里汾河生态经济带”建设力度。继续加强路网、水网、电网、通讯网建设，实施百里汾河生态文明保护区规划，推进汾河综合治理，实行严格的产业和环境准入制度，优化生态功能分区，打造优质人居环境。加大工业、农业、物流和文化旅游园区的建设力度。发挥“两区同建”工程示范作用，推动新农村和农业园区、居民社区建设。

　　(二)扎实推动供给侧改革，促进产业转型升级加大供给侧结构性改革力度，着力化解过剩产能和降本增效，努力改善产品和服务供给，稳妥处置“僵尸企业”，全力推动产业优化升级。

　　推动煤炭行业脱困转型。按照“五个一批”要求，着力化解不安全产能、不环保产能、不先进产能、不经济产能，合理发挥优势产能。围绕煤炭“六型”转变，着力推进煤矿机械化、信息化、智能化建设，延伸煤炭产业链条。今年新增生产矿井10个，煤层气钻井投用1000口。精心组织煤炭企业合理生产，严厉打击违法违规生产经营行为，加强煤炭销售，加快煤炭行业脱困转型步伐。

　　促进传统产业改造提升。焦化产业：加快向焦化并举、上下联产转变，将炼焦产能过剩劣势转化为产业竞争优势，积极探索传统焦炭转型路径。冶金产业：严控增量，优化存量，推进产品结构调整、装备水平提升、产业整合重组三大工程，向集约型、集团化、高端化的方向发展。电力产业：按照超低排放和节能改造的要求，实施兆光、大唐等电厂超低排放改造工程，加快推进晋能洪洞、蒲县宏源等低热值煤发电项目，以及安泽2×1000MW坑口电站、乡宁2×1000MW坑口电站建设，积极推进风电、光伏发电和生物质能发电项目建设。

　　培育壮大战略性新兴产业。创新产品、产业、业态，创造高质量的供给。装备制造和铸造业：发挥侯马市、翼城县、襄汾县、侯马开发区的传统铸造优势，以华翔、东方恒略、平阳重工、好利阀门等企业为重点，积极发展特色铸造产业和铸造产业集群，打造全省铸造业和装备制造基地。铝产业：抓住我省推动现代载能产业发展的机遇，加快汾西、蒲县、古县铝资源开发。重点推进汾西其亚铝业年产240万吨氢氧化铝60万吨高精铝板带箔项目、蒲县100万吨氧化铝、古县70万吨铝产业等项目建设。煤层气产业：大力实施煤矿瓦斯抽采利用全覆盖工程，实现应抽尽抽、抽采达标，积极探索煤层气多通道、多途径利用。重点推进永和—大宁—吉县—乡宁煤层气片区勘探开发。电动汽车产业：抓住我省支持电动汽车发展的政策机遇，支持电动汽车研发生产和使用推广。重点建设沃特玛新能源汽车核心技术产业园、侯马威尔捷新型太阳能电动汽车、锦江物流园汽车服务产业基地、深圳金钱潮公共充电桩等项目建设。轻工食品产业：发挥我市丰富的农产品资源优势，培育壮大一批轻工食品骨干企业。重点推进山西顶吉10万吨苹果深加工扩建项目、山西伟涛日产40万盒速冻便当等项目建设。

　　加快发展现代服务业。文化旅游业，以建设中国优秀旅游目的地城市为目标，重点推进陶寺遗址保护利用，启动建设陶寺遗址博物馆，积极申报陶寺国家考古遗址公园，撬动社会资本融入建设尧文化旅游产业园，逐步打造以塔儿山为中心的古文明旅游圈，做好“最早中国·一座都城”这篇大文章。加快壶口瀑布、人祖山、云丘山、仙洞沟、广胜寺、七里峪、乾坤湾等旅游景区景点建设，推进大槐树国家5A级旅游景区创建，全年旅游总收入增长20%以上。物流业，重点抓好山西方略保税物流、山西晋润物流园、海吉星物流园、怡和明农副产品交易物流配送中心、兴荣物流园区配送中心扩建等项目建设，加快发展第三方物流和多方物流。电子商务业，积极推行“互联网﹢物流”模式，建设淘宝特色中国临汾馆，完善马上购、宝得网等本土电商平台，促进电商与物流协同发展。积极发展新兴业态，推动互联网与煤炭、金融、现代农业、文化旅游等产业的融合。加快航空经济发展，逐步提升临汾民航机场客运、货运功能，推动航空旅游与航空运输、航空产业协同发展。

　　推进健康养老、家庭服务、房地产业等生活性服务业发展。

　　(三)全力做好“三农”和脱贫攻坚工作，促进农业发展农民增收加快发展现代农业。切实加强耕地保护，稳定粮食生产面积，推进粮食高产创建工程，提高农机化综合作业水平，确保全年粮食生产稳定在20亿公斤。以绿色、有机为发展方向，实施启动“东山以中药材、杂粮为主，平川以粮食、蔬菜、水果为主，西山以水果、杂粮为主”的三大区域板块。扎实推进“一县一业、一村一品”和农业产业园区发展，启动西山七县“果畜循环”示范区计划，全面提高特色农产品的市场竞争力。培育壮大农产品加工龙头企业，积极发展家庭农场、农民合作社，推进农业产业化经营。推动“互联网+现代农业”。

　　努力增加农民收入。把农民增收摆在突出位置，落实种粮直补、良种补贴等强农惠农政策，充分发挥政策的惠农增收效应。

　　积极推动一二三产业融合互动发展，延伸产业链条，提高农业综合效益。引导农民参 与农村人居环境改善、产业扶贫开发等农村基础设施建设，增加农民务工收入。有序推进土地流转，增加农民土地流转收益。加强新型职业农民培育，全年培训新型职业农民1.4万人。实施农民工职业技能提升计划，培训农民工6万人次，促进农村富余劳动力转移就业。推进大学生返乡创新行动、农民工返乡创业行动。

　　坚持精准扶贫、精准脱贫。按照“六个精准”和“五个一批”要求，瞄准扶贫对象，压实扶贫责任，强化政策支持，完善帮扶体系，坚决打赢脱贫攻坚战。根据西山沿黄特困地区水土流失严重、生态脆弱及农民增收困难的实际，积极探索生态补偿扶贫机制，探索解决贫困地区社会事业发展、空壳村和留守老人、儿童等问题。大力开展产业扶贫、片区开发，持续推进光伏扶贫，围绕我市光伏扶贫“两个全覆盖”的目标，继续抓好100KW村级光伏电站建设，启动建设总规模1000MW的10MW以上小型电站80座。启动实施消费扶贫，推进“互联网+消费扶贫”。同时，实施好金融扶贫、易地扶贫搬迁、教育扶贫、千村万人就业培训等扶贫重点工程，扎实开展定点扶贫和干部驻村帮扶活动。全年完成6.5万贫困人口脱贫任务。

　　(四)着力引深“三个突破”，加快培育发展新动能 发挥好科技创新的引领作用。实施创新驱动发展战略，深化产学研用合作，加强与中科院北京分院等科研院校的合作，推进企业建立院士工作站、博士后流动工作站，支持企业重点实验室、工程技术研究中心等研发中心建设，扶持建设众创空间、大学生创业园、科技孵化器等创新平台。强化企业创新主体地位和主导作用，积极培育高新技术企业。积极对接、申报国家和省级科技项目，组织实施具有创新性、引导性、示范性的市级科技项目。加大科技人才培养引进力度，积极引进高端人才和创新团队。

　　发挥好金融要素的支撑性作用。大力引进和培育金融机构，做好我市首家民营银行筹建和村镇银行普设工作，推动上海文化产权交易所和上海资源交易(服务)中心在临汾设立分支机构。推进金融创新，支持尧都农商行发展壮大，加快农信社改制工作。强化对企业上市的培育引导，推动华翔、光宇、方略、市投资集团公司、尧都农商行等企业上市，争取年内再有6家企业挂牌“新三板”。积极推进金融去杠杆化，鼓励在山西股权交易中心挂牌的企业进行股改。加强诚信体系建设，强化金融风险监测与防范，严厉打击非法集资，进一步优化金融环境。

　　发挥好民营经济的生力军作用。落实好支持民营经济健康发展的政策措施，实施民营经济“帮扶、培训、提升”三大工程，抓好企业家、专业技术人才、技术工人“三支队伍”建设。大力支持民营经济转型发展，引导民营经济进入非煤产业、新型产业、新兴产业。实施中小微企业成长工程，新创办小微企业2000户以上，新增规模以上工业企业20户以上，新认定省级小微企业创业基地1-2个。

　　(五)深化改革扩大开放，增强经济发展活力

　　全面深化各项改革。加快推进综改区建设，制订实施转型综改“十三五”方案和2016年行动计划，完成好国家、省部署的改革任务。重点深化行政审批制度改革。全面推进“六权治本”，健全完善政务服务中心管理体制，加快建设“一张网络”“两个平台”“五大功能”“四级联办”的政务服务网络平台，全面落实“两集中，两到位”。推进市县政府部门权力清单和责任清单制度。构建规范的公共资源交易体系，加强权力运行的全程跟踪监管。加快开发区扩区与园区体制机制改革。积极推进临汾、侯马开发区扩区改革，做好襄汾一区两园和洪洞赵城煤焦化深加工园区申报省级开发区工作。深化农村改革。基本完成农村土地承包经营权确权工作。加快推进农村产权流转交易市场建设，年底前县市区全部建立农村产权交易市场。加快推进农村集体产权制度、集体林权配套制度、国有林场、水权制度、小型水利工程产权和农业水价改革，健全农业保险制度。做好第三次全国农业普查工作。

　　着力扩大对外开放。积极参与“一带一路”建设，主动融入京津冀、环渤海，加强黄河金三角“三省四市”等区域交流合作。充分发挥方略保税物流中心的作用，推进外向型经济发展。

　　(六)积极推进城镇化，促进城乡统筹发展完善城市建设规划。以科学规划引领城市建设，加快编制《临汾市近期建设规划(2016-2020)》、《临汾市快速交通专项规划》、《临汾市城市地下综合管廊规划》等规划。启动百里汾河经济带、“一城三区”、侯马曲沃同城化等建设规划。着力推动各县(市、区)“十三五”近期建设规划、控制性详细规划、各类专项规划的编制工作。

　　加大中心城市建设力度。加强城市地下和地上基础设施建设，建设海绵城市，增强承载能力，提升城市品位。全力推进鼓楼南北街拓宽改造、山西师大搬迁、滨河西路与彩虹桥立交桥等市区17项重点城建工程建设，启动实施市区河西片区城市地下综合管廊工程。加快尧都区西关社区、盘龙社区、尧庙镇郭村、刘村镇涧头村、韩家庄，临 汾开发区高河店、北孝等城中村改造步伐。

　　加快推进城镇化。围绕“一带两圈多点”的城镇化布局，扎实推进12个大县城和60个重点镇建设。加大交通基础设施建设力度，吉河高速公路通车运营，霍永高速西段二期、长临高速完成年度建设任务，开工建设浮山县城至长临高速连接线、古县县城至长临高速连接线工程，完成农村公路建设500公里，完成国道209线鲁家河至十里河段吉县过境改线工程、省道洪永线车家坡至岔口段改建工程。健全城镇化推进机制，加快户籍制度改革，推行居住证制度，促进有能力在城镇稳定就业和生活的农业转移人口进城落户，全市常住人口城镇化率达到50.3%。

　　提升城市管理水平。坚持“建管并重”的原则，健全完善“网格化”、片长制、街长制等城市管理机制。推进城市管理执法体制改革，探索建立综合、快速、高效的城市综合执法体制。狠抓市容市貌和环境卫生管理，加强城市绿化。扎实开展国家卫生城市、国家园林城市创建工作，促进城市环境面貌持续改观。积极化解房地产库存，稳定房地产市场。

　　持续抓好城乡人居环境改善。实施城市人居环境改善工程，推进城市道路、水气热管网、地下综合管廊、防洪排涝等设施建设。新开工保障性住房12000套。加快推进农村人居环境改善，完成1192户地质灾害搬迁任务，做好蒲县、乡宁、洪洞等采煤沉陷区治理工作，改造农村危房11180户，新建农村老年日间照料中心90个，完成36个村生活污水治理项目，推进60个畜禽粪污治理示范点和70个病死动物无害化处理中心建设，抓好美丽宜居示范村创建工作，改善提升城乡环境面貌。

　　(七)加强生态文明建设，持续改善环境质量

　　加大环境治理力度。围绕创建国家环保模范城目标，严格落实新《环保法》、新《大气法》，强化全市环境保护网格化监管，推动与省环保厅合作协议顺利实施。集中力量打好大气、水和土壤污染防治三大战役，建立完善应对雾霾重污染天气长效机制，实行“一城三区”大气污染联防联控，建立平川7县(市、区)一体化治理机制。全面推进农作物秸秆禁烧和综合利用工作，扎实开展工业企业污染、煤烟污染、建筑施工工地扬尘、道路扬尘治理、机动车污染等专项治理，市区新增纯电动公交车200辆，投放公共自行车5055辆;整治“一城三区”燃煤小锅炉8000台。制定水污染防治计划实施细则，做好汾河生态公园水质治理试点工作，抓好重点河流生态环境综合整治工作，确保重点河流跨界断面主要污染物浓度明显下降，全市集中式饮用水源地水质达标率100%。加大土壤环境监测投入，开展污染场地环境风险评估制度建设和污染土地修复治理试点。

　　加强节能减排工作。认真实施国家节能减排财政政策综合示范项目。实行能源和水资源消耗、建设用地等总量和强度双控行动，促进节能、节水、节地、节材、节矿。扎实开展六大高耗能行业能效对标活动，有效控制电力、钢铁、建材、化工等重点行业碳排放。加大公共机构节能，推广绿色建筑。开展循环经济重点领域示范创建，推动煤矸石、粉煤灰等大宗工业固废综合利用。

　　构筑生态安全屏障。按照“三加三不减”的要求，大力开展造林绿化，深入实施林业“五大工程”，重点抓好吕梁山生态脆弱区综合治理、霍永高速通道绿化等造林绿化工程，完成营造林45.77万亩。加强对现有森林资源的管理管护。加快汾河流域生态修复步伐，实施吴村段、涝河尧乡园等4个湿地工程，浍河侯马段、黑河等4个水系整治工程。抓好涝洰河生态工程建设。推进西山七县主体功能区建设。

　　(八)抓好社会治理创新，维护社会和谐稳定毫不放松抓好安全生产。严格落实新《安全生产法》，全面推进党政同责“五个全覆盖”，按照“党政同责、一岗双责、失职追责”的要求，健全完善安全检查全留痕、安全专家会诊和安全生产标准化等三个机制，深入开展各行业、各领域安全生产专项整治，坚决打击非法违法生产经营建设行为。加大考核问责力度，严格落实安全生产“一票否决制”，实施企业重大隐患、事故挂牌督办和黑名单制度，确保全市安全生产形势持续稳定好转。

　　加强和创新社会治理。落实信访工作责任制和重点案件领导包案制度，创新城乡基层群众自治和社区治理，推进城镇社区“网格化”管理。关爱农村留守儿童、妇女和老人，加强未成年人社会保护。扎实推进“平安临汾”“法治临汾”建设，持续开展打黑除恶等严打整治行动，加强社会治安防控体系建设，提高群众的安全感和满意度。加强应急管理和防灾减灾能力建设，妥善处理各类突发事件，确保社会和谐稳定。

　　(九)坚持以人为本，着力保障和改善民生

　　大力推动文化强市建设。加强文化基础设施建设，重点推进市图书馆和市非遗传承基地项目建设，启动奥体中心、文化中心和广电中心项目，推动县级“四馆一场一院”建设。着力打造具有浓郁临汾地域特色的文艺精品，突出“尧文化”“晋国文化”“根祖文化”“丁村文化”“黄河文化”，弘扬社会主义核心价值体系。促进文化产业发展壮大，重点发展印刷包装装潢、体育健身休闲、旅游景区演艺、非物质文化遗产制造、城市数字电影等文化产业。

　　优先发展教育事业。继续抓好教育党政一把手考核和“三名”工程，启动实施优秀班主任、优质高效课、优秀教研团队“三优”培养选拔工程。积极推进义务教育均衡发展，完成永和、乡宁、大宁、隰县、蒲县5县义务教育均衡发展县验收工作。积极扶持民办教育，规范民办教育办学行为。抓好师资队伍和师德师风建设。深入推进“百校千企”工程，努力提升职业教育水平。

　　加快发展卫生计生事业。继续深化医药卫生体制改革，完善医疗卫生服务体系。加强重大疾病预防控制，加快推进分级诊疗。推进市区医疗资源整合，启动实施临汾市第三人民医院、市荣军康复医院项目。积极稳妥实施全面“两孩”政策。

　　夯实食品药品安全基础。推进可追溯体系和检验检测体系建设，强化市场监管，狠抓食品药品综合治理。扎实推进食品安全城市创建，保障群众“舌尖上的安全”。

　　推进信息惠民试点城市建设。加快信息惠民国家试点城市工程项目建设步伐，启动建设电子政务工程、云计算和大数据服务平台，实施惠民社区、教育、医疗、养老等信息工程，提升惠民服务质量。

　　完善社会保障体系。稳步提高社会保障待遇水平，健全失业保险待遇与物价水平联动调整机制。提高城乡居民医保财政补助筹资标准。实施全民参保登记计划，推动社会保障由制度全覆盖到人群全覆盖。

　　实施困难残疾人生活补贴和重度残疾人护理补贴制度。完善重特大疾病医疗救助政策。落实经济困难的高龄与失能老年人补贴以及百岁以上老年人补贴新标准。

　　在做好上述工作的同时，我们还要深入实施军民融合发展战略，支持驻临部队、武警部队、民兵预备役和人防建设;支持工会、妇联、共青团等人民团体工作;认真做好审计、统计、工商、质监、档案、水文、地震、民族宗教、外事侨务、对台和老龄等各项工作。

　　各位代表，今年，我们要继续实施“六大惠民工程”。

　　实施学前教育优质普惠工程。深入实施第二期学前教育行动计划，新改扩建农村幼儿园24所。扎实推进《临汾市城镇小区配套幼儿园规划建设与使用管理办法》的实施，规范城镇小区配套幼儿园建设。学前三年入园率达到90%。

　　实施乡村医疗服务提质工程。巩固完善基本药物制度，继续实施基层中医药服务提质工程。全面推进县乡医联体建设，强化乡镇卫生院和村卫生室服务一体化管理，提升基层医疗机构服务能力和水平。

　　实施农业强基工程。加强农田水利基本建设，大力开展土地整治、中低产田改造和高标准农田建设。全力推进大水网涉临工程，加快西山沿黄提灌工程、引沁入汾浮山供水工程建设。扎实推进“小水网”建设。

　　投资1.49亿元，实施中央财政农田水利设施建设、维修养护和膜下滴灌等项目建设。

　　全年完成实灌面积249万亩，新增和改善灌溉面积13.7万亩，完成高标准农田建设6.8万亩。

　　实施饮水安全工程。进一步加强龙祠水源地保护工作。抓好农村饮水安全巩固提升工程建设。实施规模以上集中联片供水工程的水质化验室建设、水质不达标工程的水源更新改造、水质净化与消毒设备完善配套建设。

　　实施城市综合整治工程。深入开展门店招牌、户外广告、临街建筑门面等专项治理行动，积极创建3条省级保洁示范街和3条市容市貌示范街。实施城市餐厨垃圾处理场建设工程和生活垃圾焚烧发电项目。继续实施“放心早餐工程”。

　　实施就业创业援助工程。全方位落实就业扶持措施，积极实施大学生就业促进和创业引领计划，统筹开发就业援助项目，加大对灵活就业、新就业形态的支持力度。

　　加大援企稳岗、职业技能培训、公共就业服务力度，推动创业创新，带动城乡就业。全市城镇新增就业4.3万人，转移农村劳动力就业5.1万人，城镇登记失业率控制在4.2%以内。

　　(十)加强政府自身建设，提高政府工作水平

　　推进廉洁从政。牢固树立政治意识、大局意识、核心意识、看齐意识，坚持把纪律和规矩挺在前面，认真贯彻《中国共产党廉洁自律准则》和《中国共产党纪律处分条例》，全面落实党风廉政建设“两个责任”，严格执行“一岗双责”。巩固“三严三实”专题教育成果，扎实开展“两学一做”学习教育。坚决落实中央“八项规定”和国务院“约法三章”，持续狠刹“四风”，厉行勤俭节约、反对铺张浪费，严控“三公”经费，树立为民、务实、清廉政府形象。

　　严格依法行政。自觉接受人大及其常委会的监督和政协的民主监督，认真办理人大代表议案、建议和政协提案，广泛听取各民主党派、工商联、无党派人士和各人民团体的意见。依法健全重大行政决策制度，促进决策的科学化、民主化和规范化。全面落实行政执法责任制和责任追究制度，规范行政执法行为。

　　加强作风建设。弘扬“马上就办，真抓实干”精神，继续深化“冬季行动”。强化实干意识、担当意识、执行意识，促进政府重大决策、重点工作和重要项目的落实。加强效能建设，加大问责力度，简化优化公共服务流程，提高公共服务质量和效率。

　　各位代表，实现新发展任务艰巨，创造新业绩使命光荣。让我们紧密团结在以习近平同志为总书记的党中央周围，在市委的坚强领导下，在市人大、市政协的监督支持下，凝心聚力，团结拼搏，确保“十三五”良好开局，为全面建成小康社会、实现中华民族伟大复兴中国梦而努力奋斗!
